# Natural Infection by SARS-CoV-2 in Companion Animals: A Review of Case Reports and Current Evidence of Their Role in the Epidemiology of COVID-19

**DOI:** 10.3389/fvets.2020.591216

**Published:** 2020-10-27

**Authors:** Helio Autran de Morais, Andrea Pires dos Santos, Naila Cannes do Nascimento, Louise Bach Kmetiuk, David Soeiro Barbosa, Paulo Eduardo Brandão, Ana Marcia Sá Guimarães, Christina Pettan-Brewer, Alexander Welker Biondo

**Affiliations:** ^1^Department of Clinical Sciences, Oregon State University, Corvallis, OR, United States; ^2^Department of Comparative Pathobiology, College of Veterinary Medicine, Purdue University, West Lafayette, IN, United States; ^3^Graduate College of Parasitology, Institute of Biological Sciences, Federal University of Minas Gerais, Belo Horizonte, Brazil; ^4^Department of Parasitology, Institute of Biological Sciences, Federal University of Minas Gerais, Belo Horizonte, Brazil; ^5^Department of Preventive Veterinary Medicine and Animal Health, University of São Paulo, São Paulo, Brazil; ^6^Laboratory of Applied Research in Mycobacteria, Department of Microbiology, Institute of Biomedical Sciences, University of São Paulo, São Paulo, Brazil; ^7^Department of Comparative Medicine, School of Medicine, University of Washington, Seattle, WA, United States; ^8^Department of Veterinary Medicine, Federal University of Paraná State, Curitiba, Brazil

**Keywords:** coronavirus, cats, dogs, pets, transmission

## Abstract

Severe acute respiratory syndrome coronavirus 2 (SARS-CoV-2), responsible for the coronavirus disease 2019 (COVID-19), is the causative infectious agent of the current pandemic. As researchers and health professionals are still learning the capabilities of this virus, public health concerns arise regarding the zoonotic potential of SARS-CoV-2. With millions of people detected with SARS-CoV-2 worldwide, reports of companion animals possibly infected with the virus started to emerge. Therefore, our aim is to review reported cases of animals naturally infected with SARS-CoV-2, particularly companion pets, shedding light on the role of these animals in the epidemiology of COVID-19.

## Introduction

SARS-CoV-2, or severe acute respiratory syndrome coronavirus 2 (1), is an emergent, zoonotic pathogen first identified in China in late 2019 (2, 3). This enveloped, positive-sense single-stranded RNA virus is a novel *Betacoronavirus* (4) with phylogenetic proximity to SARS-CoV-1 (2, 3). In humans, SARS-CoV-2 can cause asymptomatic infections to severe atypical pneumonia that can lead to death. Given its rapid spread in different countries, the disease, named COVID-19 (i.e., Coronavirus Disease 2019), was declared a Public Health Emergency of International Concern by the World Health Organization (WHO) in January 2020 (5). In only 2 months, the virus spread to all continents, except Antarctica, and in March 2020, COVID-19 was characterized by WHO as a pandemic (5). On August 3, 2020, SARS-CoV-2 has been present in 213 countries or regions and detected in at least 17 million people, while 690,000 individuals have succumbed to the disease (6, 7).

According to the United States Agency for International Development (USAID), nearly 75% of all new emerging or re-emerging infectious diseases of the last century originated in animals, such as HIV, Ebola, avian influenza, and swine influenza (8). Accordingly, the initial epicenter of SARS-CoV-2 was linked to possible contact with wild animals sold at wholesale seafood and exotic animal markets of Huanan, Wuhan, Hubei province, China (5). Analysis of complete genome sequences of the new coronavirus isolated from patients during the initial stage of the outbreak in Wuhan showed only about 79% identity with SARS-CoV-1 (severe acute respiratory syndrome coronavirus 1), identified in China in 2002 (9, 10), and 50% identity with MERS-CoV (Middle East respiratory syndrome coronavirus), identified in Saudi Arabia in 2012 (4, 11). Interestingly, it revealed 96% identity with a bat coronavirus (BatCoV) found in *Rhinolophus affinis* (horseshoe bat), named RaTG13, sampled in Yunnan province, China, in 2013 (12), and 91.02% identity with a coronavirus obtained from pangolins (*Manis javanica*) (13, 14). This close phylogenetic relatedness of SARS-CoV-2 to non-human coronaviruses, in the absence of a known ancestral virus sample, strongly suggests a viral host jump from wildlife to humans, most likely from bats (12, 13, 15). More detailed genomic analyses indicate that SARS-CoV-2 is a product of natural selection rather than laboratory manipulation and that an animal source was likely involved in the initial cases of human infections associated with the Huanan market (15). Because contact between humans and bats is a rare event, it is also possible that a susceptible intermediate host species may have participated in the epidemiology of SARS-CoV-2, similar to what was observed with SARS-CoV-1 and MERS-CoV (16, 17).

Coronaviruses (CoVs) tend to be species specific when it comes to hosts, which is determined by the interaction of the virus with specific host cell receptors (18). The spike protein, a protruding glycoprotein of the membrane of CoV virions, mediates host cell adhesion and membrane fusion (18). The amino acid sequence of the spike protein is what defines its ability to interact with different host cell receptors. For three of the human coronaviruses, HCoV-NL63, SARS-CoV-1, and SARS-CoV-2, the angiotensin-converting enzyme 2 (ACE-2) has been identified as the cell receptor with which the spike protein interacts (19–22). For the adhesion to occur properly, researchers have identified 69 amino acid residues at the receptor binding domain (RBD) of the spike protein that are key for its interaction with ACE-2 (22). Although both SARS-CoV-2 and SARS-CoV-1 use the same receptor, their RBD is different at five out of six important amino acid residues. Surprisingly, only one of these residues was identical between SARS-CoV-2 and the BatCoV RaTG13, while all six are identical between SARS-CoV-2 and the pangolin CoV (23). Thus, although the BatCoV RaTG13 is the closest relative to SARS-CoV-2 at the whole-genome level, the RBD residues critical for receptor interaction are actually identical to pangolin CoVs (23). This finding is supportive evidence of a natural selection process during a viral host jump from animals to humans.

As detailed above, the amino acid sequence of ACE-2 is a determining factor of the host species range affected by SARS-CoV-2. During the search for an animal model of COVID-19, bioinformatic predictions and previous studies with SARS-CoV-1 indicated that non-human primates, ferrets, hamsters, and domestic cats were possible animal candidates to be explored (24–28). Accordingly, experimental SARS-CoV-2 infection and clinical sign development have been successfully accomplished in non-human primates, ferrets, and golden Syrian hamsters (*Mesocricetus auratus*) (27, 29–33). Experimental infection was also successful in cats, but the animals developed no clinical signs (31, 34). In dogs, the intranasal inoculation of SARS-CoV-2 in 3-month-old beagles resulted in only two out of four experimentally infected animals developing neutralizing antibodies and no detectable viral RNA in organ tissues of one euthanized animal 4 days postinfection. Viral RNA was sporadically detected in the feces of these dogs a few days postinfection (31). This same study shows that experimental infection of SARS-CoV-2 was not successful in pigs, ducks, and chickens (31). Taken together, these results raise the possibility that companion animals, particularly cats and hamsters, may get infected with SARS-CoV-2 outside experimental laboratory conditions. As previously shown in a review on cell, tissue, and animal models for SARS-CoV-2 infection, non-human primates may be used for human clinical tests, while primary cell culture, primary tissue explants, and organoids may be applied for other human and animal approaches (35).

With millions of people detected with SARS-CoV-2 worldwide, reports of companion animals possibly infected with the virus started to emerge. These animals were frequently owned by individuals with confirmed or suspected SARS-CoV-2 infection, raising concerns that an anthropozoonotic transmission occurred. Therefore, our aim is to review reported cases of animals naturally infected with SARS-CoV-2, particularly companion pets, shedding light on the role of these animals in the epidemiology of COVID-19.

## A Brief Overview of Coronaviruses

Members of the *Coronaviridae* family have a positive-sense single-stranded RNA genome varying from 26 to 32 kilobases, the largest viral RNA genomes ever described. CoVs are enveloped viruses and identified in several species of birds and mammals, including humans. The *Coronaviridae* family is composed of two subfamilies (*Letovirinae* and *Orthocoronavirinae*) and four genera (*Alphacoronavirus, Betacoronavirus, Gammacoronavirus*, and *Deltacoronavirus*) found in the subfamily *Orthocoronavirinae*. The organization and expression of their genome are very similar, with 15 to 16 non-structural proteins (nsp1 to nsp16, with nsp1 being absent in *Gammacoronavirus*), codified by ORF1ab at the 5′ end, and four to six structural proteins, hemagglutinin-esterase (HE, found in some betacoronaviruses) spike (S), envelope (E), membrane (M), and nucleoprotein (N), which are codified by ORFs at the 3′ end of the genome (36, 37).

In humans, CoVs primarily cause infections of the upper respiratory and gastrointestinal tracts, with clinical manifestations ranging from asymptomatic to severe or lethal (38). Seven CoV strains are able to infect humans: HCoV-NL63, HCoV-229E (*Alphacoronavirus*), HCoV-OC43, HCoV-HKU1, SARS-CoV-1, MERS-CoV, and more recently, SARS-CoV-2 (all these in the *Betacoronavirus* genus) (36, 38). HCoV-NL63, HCoV-229E, HCoV-OC43, and HCoV-HKU1 are distributed globally (39, 40), with seasonal and geographic variations. These are low-pathogenic CoVs associated with a variety of mild upper respiratory tract infections, occasionally affecting the lower respiratory tract, leading to pneumonia, bronchiolitis, or both (40–44). Nonetheless, in the last two decades, highly pathogenic, zoonotic CoVs emerged. These are SARS-CoV-1, which emerged in China in 2002 (9, 10); MERS-CoV (11), which was first detected in Saudi Arabia in 2012 (11); and SARS-CoV-2 identified in China in 2019 (2, 3). These CoVs are highly pathogenic and may cause lethal disease, with variable mortality rates of about 10% for SARS-CoV-1, 34% for MERS-CoV, and from 1 to 7% for SARS-CoV-2. The epidemic of SARS-CoV-1 affected 26 countries, and more than 8,000 cases were reported, while MERS-CoV was identified in 27 countries, with more than 80% of the 2,494 cases reported in Saudi Arabia (10, 45). Currently, SARS-CoV-1 is not detected in any region of the world, and MERS-CoV cases are sporadically reported in Saudi Arabia (10, 45). SARS-CoV-2, on the other hand, is currently at epidemic peaks in many regions, with exponential growth of case numbers and fatalities globally (7).

Coronaviridae species affecting host species other than humans have been reported, causing respiratory, gastrointestinal, liver, kidney, or neurological diseases in a variety of domestic and wild animals, with no human infection by these coronaviruses ever reported. Among companion animals, canine coronavirus (CCoV) and feline coronavirus (FCoV) belong to the species *Alphacoronavirus* 1 with two serotypes (I and II), each occurring as either a low-virulence biotype that causes mild to silent enteric infectious and high-virulence, pantropic biotypes in dogs (a CCoV-IIa lineage) and cats (feline infectious peritonitis virus) (46, 47). In addition, a betacoronavirus named canine respiratory coronavirus has been associated with respiratory disease in dogs (48).

Among large animals, calves and adult cattle are susceptible to enteric and respiratory disease after infection by bovine coronavirus (a *Betacoronavirus*), while another betacoronavirus, equine coronavirus, has been associated with enteric disease in horses (49, 50). Avian coronavirus (a *Gammacoronavirus*) has chickens as a natural host, infecting the trachea, lungs, kidneys, reproductive tract, and intestines in broilers, layers, and breeders with a wide range of serotypes (51, 52). Moreover, swine acute diarrhea syndrome coronavirus (SADS-CoV), first reported in 2017, together with porcine epidemic diarrhea virus (PEDV) and transmissible gastroenteritis virus (TGEV) are alphacoronaviruses that cause highly lethal enteric disease in pigs (53). Porcine deltacoronavirus (PDCoV) and the betacoronavirus porcine hemagglutinating encephalomyelitis virus (PHEV) also use pigs as hosts (54, 55).

## ACE-2 Similarity Among Domestic Animals and Humans

To understand how certain domestic animal species may be infected with SARS-CoV-2, it is crucial to investigate the underlying reason for the ability of the virus to enter host cells and establish infection. Current knowledge of the SARS-CoV-2 pathogenesis indicates that such event is made possible by the interaction between SARS-CoV-2 and the host ACE-2 protein, which acts as a receptor for viral adherence and membrane fusion (19–22). Supplementary Data and Table 1 show a multiple protein sequence alignment of ACE-2 of human, main domestic, and laboratory animal species and cross-species identity of the 22 amino acids of ACE-2 that physically interact with SARS-CoV-2 (22), respectively. Among putative pet animals, golden Syrian hamsters, cats, and rabbits diverge in only 3 of the 22 amino acids of ACE-2 responsible for the interaction with SARS-CoV-2, while dogs diverge in five amino acids (Supplementary Data and Table 1). However, the whole-protein sequence of ACE-2 of golden Syrian hamsters showed higher sequence similarity and phylogenetic relatedness to human ACE-2 than the rabbit and cat ACE-2 (Supplementary Figure 1). Whether at the whole-protein level or at the 22 interaction-defining amino acids, this sequence may be determinant of a successful infection, along with the expression of ACE-2 protein in different tissues and yet to be unraveled alternative receptors of SARS-CoV-2 in host cells. The link between structural properties of ACE-2 orthologs to SARS-CoV-2 spike protein has been already investigated (56). In this study, non-conservative mutations in several ACE-2 amino acid residues have been associated with interrupted key polar and charged viral spike protein contacts, which may decrease the susceptibility to SARS-CoV-2 infection across different animal species. In addition, structural analysis of amino acid residues has suggested that changes in amino acid positions 30 and 83 may affect structural interaction of ACE-2 and SARS-CoV-2 RBD, differentiating non-susceptible from susceptible species (57).

**Table 1:** Cross-species identity of the 22 amino acids of the angiotensin-converting enzyme-2 (ACE-2) identified as directly involved in the physical interaction of ACE-2 and severe acute respiratory syndrome coronavirus 2 (SARS-CoV-2), as defined by Shang et al. (22).

	**Amino acid position in the human ACE-2**																						
**Host**	**19**	**24**	**27**	**28**	**31**	**34**	**35**	**37**	**38**	**41**	**42**	**45**	**79**	**82**	**83**	**325**	**329**	**330**	**353**	**354**	**355**	**357**	**Total**
Human	S	Q	T	F	K	H	E	E	D	Y	Q	L	L	M	Y	Q	E	N	K	G	D	R	22/22
Rhesus monkey	S	Q	T	F	K	H	E	E	D	Y	Q	L	L	M	Y	Q	E	N	K	G	D	R	22/22
Chimpanzee	S	Q	T	F	K	H	E	E	D	Y	Q	L	L	M	Y	Q	E	N	K	G	D	R	22/22
Syrian hamster	S	Q	T	F	K	Q	E	E	D	Y	Q	L	L	N	Y	Q	E	N	K	G	D	R	20/22
Domestic cat	S	L	T	F	K	H	E	E	E	Y	Q	L	L	T	Y	Q	E	N	K	G	D	R	19/22
Cow	S	Q	T	F	K	H	E	E	D	Y	Q	L	M	T	Y	Q	D	N	K	G	D	R	19/22
Sheep	S	Q	T	F	K	H	E	E	D	Y	Q	L	M	T	Y	Q	D	N	K	G	D	R	19/22
Rabbit	S	L	T	F	K	Q	E	E	D	Y	Q	L	L	T	Y	Q	E	N	K	G	D	R	19/22
Chinese hamster	S	Q	T	F	K	Q	E	E	D	Y	Q	L	L	N	Y	Q	G	N	K	G	D	R	18/22
Pangolin	S	E	T	F	K	S	E	E	E	Y	Q	L	I	N	Y	Q	E	N	K	H	D	R	17/22
Domestic dog	S	L	T	F	K	Y	E	E	E	Y	Q	L	L	T	Y	Q	G	N	K	G	D	R	17/22
Horse	S	L	T	F	K	S	E	E	E	H	Q	L	L	T	Y	Q	E	N	K	G	D	R	17/22
Pig	S	L	T	F	K	L	E	E	D	Y	Q	L	I	T	Y	Q	N	N	K	G	D	R	17/22
Ferret	S	L	T	F	K	Y	E	E	E	Y	Q	L	H	T	Y	E	Q	N	K	R	D	R	14/22
Mice^*^	S	N	T	F	N	Q	E	E	D	Y	Q	L	T	S	F	Q	A	N	H	G	D	R	14/22
G. horseshoe bat^**^	S	L	K	F	D	S	E	E	N	H	Q	L	L	N	F	E	N	N	K	G	D	R	13/22
Chicken	S	-	T	F	E	V	R	E	D	Y	E	L	N	R	F	E	T	N	K	N	D	R	12/22

* *Mus musculus*.

** *Greater horseshoe bat. Total = number of identical amino acid residues compared to the human ACE-2 reference amino acids. Accession numbers of protein sequences: NP_001358344.1 (human, Homo sapiens); NP_001116542.1 (pig, Sus scrofa); NP_001019673.2 (cow, Bos taurus); NP_001158732.1 (domestic dog; Canis lupus familiaris); NP_001034545.1 (domestic cat, Felis catus); XP_416822.2 (chicken; Gallus gallus); XP_011961657.1 (sheep; Ovis aries); XP_003503283.1 (Chinese hamster; Cricetulus griseus); NP_001123985.1 (mice, Mus musculus); XP_002719891.1 (rabbit; Oryctolagus cuniculus); XP_016798468.1 (chimpanzee, Pan troglodytes); NP_001129168.1 (Rhesus monkey; Macaca mulatta); NP_001297119.1 (European domestic ferret, Mustela putorius furo); XP_005074266.1 (golden Syrian hamster, Mesocricetus auratus); XP_001490241.1 (horse, Equus caballus); XP_017505752.1 (pangolin, Manis javanica); XP_032963186.1 (greater horseshoe bat; Rhinolophus ferrumequinum). Whole-protein sequence alignment can be found in the Supplementary Data. Amino acid residues are the ones in contact with the receptor binding domain (RBD) of SARS-CoV-2 and directly involved in the RBD–ACE-2 binding. Positions were retrieved from Shang et al. (22). Overall protein identity/similarity against human ACE-2 of chimpanzee = 99.0/99.4%; Rhesus monkey = 94.9/97.5%; golden Syrian hamster = 84.5/91.7%; domestic cat = 81.7/88.3%; cow = 81.0/90.6%; sheep = 81.7/90.8%; rabbit = 85.2/92.8%; Chinese hamster = 84.3/91.6%; pangolin = 84.8/91.3%; domestic dog = 83.5/91.8%; horse = 86.8/93.4%; pig = 81.4/90.7%; ferret = 82.6/91.6%; mice = 82.1/89.6%; greater horseshoe bat = 81.5/90.3%; chicken = 65.6/79.3%. Pairwise protein identities and similarities were calculated using the Needleman–Wunsch algorithm from the European Bioinformatics Institute (EMBL-EBI) available at https://www.ebi.ac.uk/Tools/psa/emboss_needle/ using default parameters*.

## SARS-CoV-2 in Animals

### Dogs

The first report of SARS-CoV-2 infection in dogs occurred in Hong Kong, China, by the Hong Kong Agriculture, Fisheries, and Conservation Department (AFCD) (58) (Table 2). A 17-year-old male Pomeranian with several comorbidities was asymptomatic and quarantined on February 26, 2020, after its owner was diagnosed with COVID-19 (58, 59). On March 18, an asymptomatic 2.5-year-old male German shepherd dog tested positive for SARS-CoV-2 by RT-qPCR in nasal and oral swabs; the two dogs had detectable antibodies against SARS-CoV-2 (60, 61). In addition, viral sequences were identical to the virus identified in the respective owner cases, suggesting human-to-animal transmission (72). On June 1, in the Netherlands, neutralizing antibodies against SARS-CoV-2 were detected in an 8-year-old American bulldog with breathing distress, with a COVID-19-positive owner (62). The animal was euthanized due to clinical worsening (71). In New York State, Richmond County, USA, two dogs tested positive to anti-SARS-CoV-2 antibodies (62). One dog showed signs of respiratory illness and severe lethargy associated with hemolytic anemia (62). The other dog was asymptomatic. The owner of the two dogs tested positive for COVID-19 (62). Both dogs are recovering (62). In May 2020, a 7-year-old male German Shepherd tested positive for SARS-CoV-2 by RT-qPCR, after 6 weeks with breathing distress (73). On July 11, 2020, the animal died with a diagnosis of lymphoma, which may have been a confounding cause for the respiratory signs (73).

**Table 2:** Reports of SARS-CoV-2 natural infection in animals worldwide, to date.

**Test date**	**Species**	**Location**	**Clinical signs (%)**	**Sample**	**Test**	**Positive/total animals (%)**	**References**
February 26, 2020	Dog	Hong Kong/China	No	Oral and fecal swab Serum samples	RT-qPCR PRN	1/1 (100.0)	(58, 59)
March 18, 2020	Dog	Hong Kong/China	No	Nasal and oral swabs Serum samples	RT-qPCR PRN^*^	1/1 (100.0)	(60, 61)
March to April, 2020	Cat	Hong Kong/China	No	Nasal, oral, and fecal swabs	RT-qPCR	1/17 (5.9)	(62, 63)
March 18, 2020	Cat	Belgium	Yes	Vomit and feces samples	RT-qPCR	1/1 (100.0)	(64)
April 4, 2020	Tiger	New York City/USA	Yes	Nasal and oral swabs	RT-qPCR	4/5 (80.0)	(65)
April 4, 2020	Lion	New York City/USA	Yes	Nasal and oral swabs	RT-qPCR	3/3 (100.0)	(66)
April, 17 2020	Cat	France	Yes	Rectal swab	RT-qPCR; MIA^**^	1/22 (4.5)	(67)
April, 22 2020	Cat	New York State/USA	Yes	Nasal swabs	RT-qPCR	2/2 (100.0)	(68, 69)
April 26, 2020	Mink	Netherlands	Yes	Nasal, oral, and rectal swabs	RT-qPCR	7/7 (100.0)	(70)
July 1, 2020	Dog	Netherlands	Yes	Serum samples	Serological test	1/1 (100.0)	(71)
July 2, 2020	Dog	New York State, Richmond County/USA	Yes (1/2; 50.0)	Serum samples	Serological test	2/2 (100.0)	(62)

* *PRN, plaque reduction neutralization test*.

** *MIA, microsphere immunoassay*.

### Cats

Also, in Hong Kong, nasal and oral swab and fecal samples from a clinically healthy cat tested positive for SARS-CoV-2 by RT-qPCR (74). The owner had been hospitalized with COVID-19 (63). Until April 15, 2020, the Hong Kong Agriculture, Fisheries, and Conservation Department tested 17 cats from guardians positive for COVID-19, and only the cat mentioned above was positive (62). In mid-March 2020, in Belgium, viral RNA from SARS-CoV-2 was detected in samples of vomit and feces of a cat with diarrhea, vomiting, and dyspnea, using RT-qPCR (64). Despite the animal's guardian being infected with COVID-19, it was not possible to establish the level of identity between the genomic sequences of SARS-CoV-2 present in the cat and human (64). This cat showed clinical improvement 9 days after the onset of symptoms (64).

On April 22, the OIE, the Centers for Disease Control and Prevention (CDC), and the United States Department of Agriculture (USDA) reported that two cats from the New York I State in the USA, both presenting sneezing and nasal discharge, tested positive for SARS-CoV-2 by RT-qPCR (68, 69). One cat is a 5-year-old Devon Rex, from Orange County, and the owner was positive for COVID-19 (75). The clinical signs in the cat appeared after the owner showed COVID-19-compatible symptoms (75). Another cat in the same household remained asymptomatic but was not tested for the presence of the virus (75). The second positive 4-year-old cat was from Long Island (Nassau County) with outdoor access (75). The animal has presented respiratory signs and lethargy and tested positive to SARS-CoV-2 RNA by quantitative RT-PCR. Three of five households have shown clinical signs related to COVID-19 but were not tested, and the cat is presumed to have been infected by someone at home or by a virus carrier (75). The latest laboratory tests on the two cats have shown that they are clearing the infection and will likely have full recovery (75).

On March 18, a case of a Belgian cat with a breathing problem, vomiting, and diarrhea was reported, with SARS-CoV-2 detected by RT-qPCR in vomit and feces samples (76). On April 17, a 9-year-old female cat of European breed from France was tested positive to SARS-CoV-2 RNA by RT-qPCR on a rectal swab. The animal showed clinical signs, such as anorexia, vomiting, and cough, 17 days after its owner has tested positive to COVID-19. Antibodies against SARS-CoV-2 have been detected in two serum samples taken 10 days separately. In addition, sequence analysis of cat SARS-CoV-2 has shown that it belongs to the phylogenetic clade A2a, similar to the French human SARS-CoV-2 (67). In June, one cat from Minnesota and another one from Illinois, USA, tested positive for SAR-CoV-2, confirmed by USDA's National Veterinary Services Laboratories (77).

It is not surprising that cats develop clinical signs; SARS-CoV-2 penetrates the cell by binding to the ACE-2 receptor, and the ACE-2 receptor in cats has high homology with the human receptor (78–80), as shown above. The coronavirus that caused the SARS epidemic (SARS-CoV-1) in 2003 also uses the ACE-2 receptor to enter cells (81). Cats are susceptible to experimental infection with the SARS-CoV-1 and also became naturally infected during the SARS epidemic in 2003 (81, 82). Recently, cats were experimentally inoculated intranasally with high doses of SARS-CoV-2 (31). The animals showed no clinical signs, developed neutralizing antibodies, and eliminated viral RNA in the feces. At necropsy, infectious virus was found in the nasal turbinates, soft palate, tonsils, trachea, and lungs (31). Experimentally infected cats transmitted the disease by air particles to susceptible cats (31). Moreover, an experimental study has shown that SARS-CoV-2 was transmitted by virus-inoculated cats to cats with no previous infection, after cohoused contact. After 24 days of inoculation, all the cats showed IgG antibody titers ranging from 5,120 to 20,480. Since no clinical signs were reported in this study, the authors speculate that cats may be a silent intermediate host of SARS-CoV-2 (34).

### Other Animals

Experimental infection with SARS-CoV-2 is also possible in hamsters, ferrets, rhesus macaques (*Macaca mulatta*), cynomolgus monkeys (*Macaca fascicularis*), and African green monkeys (*Chlorocebus sabaeus*) (31–33, 83–86). *Callithrix jacchus* monkeys have been resistant to SARS-CoV-2 experimental infection, when compared with *M. fascicularis* and *M. mulatta* (87). Experimental studies with rhesus macaques have shown mild disease as frequently observed in human cases (83) and suggest that primary SARS-CoV-2 infection protects against reinfection throughout early recovery days (88).

In hamsters and ferrets, transmission occurred to other susceptible animals by air (31, 32). To date, there are no reports of natural cases of SARS-CoV-2 infection in hamsters or ferrets (31, 32). The Hong Kong Agriculture, Fisheries, and Conservation Department tested two hamsters from guardians positive for SARS-CoV-2, and both were negative (62). Under experimental conditions, Syrian hamsters (*M. auratus*) have been successfully infected by SARS-CoV-2, and transmission between cohoused animals was observed by direct or indirect contact with blood, feces, saliva, and tears (27).

On April 4, 2020, the US Department of Agriculture (USDA) announced that samples from a 4-year-old female Malayan tiger at the Bronx Zoo in New York City tested positive for SARS-CoV-2 by RT-qPCR (89). The swab samples were collected and tested after two Malayan tigers, three Siberian tigers, and three African lions showed respiratory signs for a week (65, 89). On April 17, the OIE confirmed that one of the African lions tested positive for SARS-CoV-2 by RT-qPCR (66). Later on, all these animals and one asymptomatic Siberian tiger tested positive for SARS-CoV-2 by RT-qPCR of stool samples (90). The five positive tigers live separately in the same enclosure (90). The three lions live in an enclosure in another zoo area, and they occasionally interacted (90). The Bronx Zoo also has one Malayan tiger and two Siberian tigers living in a distant enclosure (91). These three tigers showed no clinical signs (91). SARS-CoV-2 was identified and characterized in a Malayan tiger (92). The seven symptomatic animals have improved and are expected to fully recover (91, 93). In addition, SARS-CoV-2 characterization has shown distinct viral sources for tigers and lions, with similarities between tiger and zookeeper viruses suggesting human–animal transmission, but no identified viral source was found for the infection in lions (94).

On April 26, 2020, the Dutch Ministry of Agriculture, Nature, and Food Quality communicated SARS-CoV-2 outbreak in two mink (*Neovison vison*) farms, after respiratory disease and increased mortality (70, 95). Infection by SARS-CoV-2 has been reported in minks on a farm with 13,000 minks (95). Additional infections were identified on a second farm with 7,500 adult minks (96). Three minks with gastrointestinal and respiratory signs were euthanized (70). Samples of manure, air, and dust collected from the vicinity of the farm are being tested for the presence of the virus (95, 96). Cats from the farms will also be tested (95). At least one worker tested positive for SARS-CoV-2 in both farms (70). It is not surprising that minks are susceptible because they are from the same family (*Mustelidae*) as ferrets (*Mustela putorius furo*), and ferrets can be experimentally infected with SARS-CoV-2 and transmit the disease to other ferrets by direct or indirect contact (32, 33). The infection in minks appears to be a case of human-to-animal infection, once viral sequences of two farms were related to human being sequences, but in separate introductions (70). In addition, since March 2020, rabbit farms near infected visons have been investigated by the Dutch Ministry of Agriculture, Nature, and Food Quality due to possible susceptibility to SARS-CoV-2 (97).

Experimental transmission study has shown that pigs (*Sus scrofa*) and chickens (*Gallus gallus*) were not susceptible to SARS-CoV-2, since none of the animals seroconverted and all samples were negative for viral RNA after intranasal inoculation (98). On the other hand, fruit bats (*Rousettus aegyptiacus*) have presented virus replication detected by RT-PCR, *in situ* hybridization (ISH), and immunohistochemistry (IHC) associated with mild rhinitis (98).

## Discussion

It is important to emphasize that there is no transmission of SARS-CoV-2 from pets to humans to date and that transmission from people to pets is rare. In a study carried out by the Pasteur Institute (France) published in April 2020, 21 domestic animals were tested, including 9 cats and 12 dogs that lived in close contact with their guardians, a total of 20 veterinary students in France (99). Among the students, two tested positive for SARS-CoV-2 by RT-PCR, and 11 out of 18 showed clinical signs of COVID-19 (99). Among the animals, three cats showed respiratory and gastrointestinal symptoms (99). Despite the proximity to infected guardians, no dog or cat tested positive for SARS-CoV-2 by RT-PCR nor showed antibodies to SARS-CoV-2 in an immunoprecipitation assay (99). Despite the low sampling, the study suggested that the transmission rate of SARS-CoV-2 between humans and pets under natural conditions is probably very low, below a reproduction number of 1 (99). So far, there is no epidemiological study with a large number of animals that allows estimating the percentage of dogs and cats in contact with people with COVID-19 that excrete the virus or develop antibodies.

Cats are known to be more susceptible to experimental infection with SARS-CoV-2 than dogs (31). In 2016, 21% of New York State households in the USA had cats with an estimated total of 2,841,000 cats, and 21% of households had an average of 1.7 cats per house (100). As of May 9, 2020, the New York State had 333,000 confirmed cases of COVID-19 (101). If those cases were from different households, ~103,000 cats would have been exposed to patients with COVID-19, and only 2 of these 103,000 cats tested positive for SARS-CoV-2 (101). Cat population in the area that would have been exposed to the virus was estimated. Despite the impossibility of knowing how many cats have been tested, evidences have shown that clinical disease may be rare in cats. This suggests that transmission from people to animals is really rare.

There are two serological surveys with cats, one of which also includes dogs (102). In an unpublished study, 11 of 102 cats had neutralizing antibodies against SARS-CoV-2, suggesting that under natural conditions, cats exposed to SARS-CoV-2 develop antibodies (103). These samples were obtained after the outbreak of COVID-19 in Wuhan, China (103). In another serological study also in the Wuhan region, antibodies to SARS-CoV-2 were investigated in 35 animal species (102). The sampling included 15 pet dogs, 99 street dogs, 66 pet cats, and 21 street cats (102). Close contact with a patient with COVID-19 was confirmed for at least three dogs in this study (102). None of the dogs and cats had antibodies against SARS-CoV-2 (102). It is possible that the infected companion animals reported so far were in close contact with humans emanating high viral loads of SARS-CoV-2, had comorbidities or increased susceptibility to the virus, or had a combination of these factors. It is assumed that the risk of animals is greater at the beginning of the disease in people because this is the time when the viral load is higher (104).

One must take into account that if a sustained transmission naturally establishes among individuals of the same species, SARS-CoV-2 might be led by natural selection to achieve a higher fitness in this new species; the consequences of this event remain unclear.

Many residents at the original epicenter of the Wuhan outbreak were forced to leave their pets behind when authorities removed people from their homes (105). Reports suggest that owners left enough food and water for their pets to last for some days (106). Several weeks later, many residents had not yet returned home. In China, animal welfare organizations estimate that in Hubei alone, tens of thousands of cats and dogs have been left behind, facing hunger and death (105). The risk of pet abandonment may increase due to reports of SARS-CoV-2 in dogs and cats, associated to lack of reliable information ruling out dogs and cats as a source of infection. To investigate whether the abandonment of domestic cats protects people against SARS-CoV-2 infections, a computer model was created that simulates a small community of human households and cats (107). A different number of cats were set free during the simulations, and the total number of infected people was recorded (107). In the simulations, cats were chosen randomly, regardless of whether they were positive or not, to simulate people in panic abandoning their cats out of fear (107). After 2,000 simulations, it was concluded that the number of infected people varies significantly according to the number of abandoned cats (107). When no cat was set free, 51 people on average were infected (107). For one cat set free, 55 people were infected. For five set free, 62 were infected. For 10 abandoned cats, 76 people in the community were infected. This model suggests that abandoning cats can increase the risk of infection among people. The model is still rather superficial, and some of the assumptions are questionable (107). The likelihood of infection from one cat to another cat was considered to be the same as that of people to people, while transmission between different species is approximately half the probability (107). These values are probably overstated when it comes to transmission between cats and transmission interspecies (107). Still, this is a good example of a simulation that can assist in risk assessment and decision making and in the effect of changing some parameters on the incidence of new cases in human patients (107).

## Author Contributions

HM, AS, AG, DB, and AB contributed to the conception and design of the study. HM, AS, LK, DB, and AB wrote the first draft of the manuscript. HM, AS, NN, LK, DB, PB, AG, CP-B, and AB wrote sections of the manuscript. All authors contributed to the manuscript revision and read and approved the submitted version.

## Conflict of Interest

The authors declare that the research was conducted in the absence of any commercial or financial relationships that could be construed as a potential conflict of interest.
