# Supplementary material for: Natural Infection by SARS-CoV-2 in Companion Animals: A Review of Case Reports and Current Evidence of Their Role in the Epidemiology of COVID-19
Source: Front Vet Sci. 2020 Oct 27;7:591216. doi: 10.3389/fvets.2020.591216 (PMC7652926; doi:10.3389/fvets.2020.591216)
Supplement: Supplementary file 1 [file Data_Sheet_1.pdf]

frontiers

Supplementary Material

1 **Supplementary Data.** Multiple sequence alignment of protein sequences of ACE-2 (angiotensin converting enzyme 2) of human, domestic and laboratory animals, and selected wild animal species. Multiple sequence alignment was performed using Clustal Omega tool available at https://www.ebi.ac.uk/Tools/msa/clustalo/ from the European Bioinformatics Institute (EMBL-EBI) usin g default parameters. In bold are highlighted conserved amino acid residues of the 22 amino acid positions of the human ACE-2 that have been identified to interact with the receptor binding domain of the SARS-CoV-2 (Severe Acute Respiratory Syndrome Coronavirus 2) (Shang et al., 2020). Accession numbers of protein sequences: NP_001358344.1 (Human, *Homo sapiens*); NP_001116542.1 (Pig, *Sus scrofa*); NP_001019673.2 (Cow, *Bos taurus*); NP_001158732.1 (Domestic dog; *Canis lupus familiaris*); NP_001034545.1 (Domestic cat, *Felis catus*); XP_416822.2 (Chicken; *Gallus gallus*); XP_011961657.1 (Sheep; *Ovis aries*); XP_003503283.1 (Chinese hamster; *Cricetulus griseus*); NP_001123985.1 (Mice, *Mus musculus*); XP_002719891.1 (Rabbit; *Oryctolagus cuniculus*); XP_016798468.1 (Chimpanzee, *Pan troglodytes*); NP_001129168.1 (Rhesus monkey; *Macaca mulatta*); NP_001297119.1 (European domestic ferret, *Mustela putorius furo*); XP_005074266.1 (Golden Syrian hamster, *Mesocricetus auratus*); XP_001490241.1 (Horse, *Equus caballus*); XP_017505752.1 (Pangolin, *Manis javanica*); XP_032963186.1 (Greater Horseshoe bat; *Rhinolophus ferrumequinum*). Overall protein identity/similarity against human ACE-2 of Chimpanzee = 99.0%/99.4%; Rhesus monkey = 94.9%/97.5%; Golden Syrian hamster = 84.5%/91.7%; domestic cat = 81.7%/88.3%; cow = 81.0%/90.6%; sheep = 81.7%/90.8%; rabbit = 85.2%/92.8%; Chinese hamster = 84.3%/91.6%; pangolin = 84.8%/91.3%; domestic dog = 83.5%/91.8%; horse = 86.8%/93.4%; pig = 81.4%/90.7%; ferret = 82.6%/91.6%; mice = 82.1%/89.6%; greater horseshoe bat = 81.5%/90.3%; chicken = 65.6%/79.3%. Pairwise protein identities and similarities were calculated using Needleman-Wunsch algorithm from the European Bioinformatics Institute (EMBL-EBI) available at https://www.ebi.ac.uk/Tools/psa/emboss_needle/ using default parameters.

Homo sapiens	MSSSSWLLLSLVAVTAAQ**S**TIEE**Q**AK**TF**LD**K**FN**HE**A**ED**LF**YQ**SS**L**ASWNYNTNITEENVQ
Macaca mulatta	MSGSSWLLLSLVAVTAAQ**S**TIEE**Q**AK**TF**LD**K**FN**HE**A**ED**LF**YQ**SS**L**ASWNYNTNITEENVQ
Pan troglodytes	MSGSSWLLLSLVAVTAAQ**S**TIEE**Q**AK**TF**LD**K**FN**HE**A**ED**LF**YQ**SS**L**ASWNYNTNITEENVQ
Rhinolophus sp	MSGSSWLLLSLVAVTAAQ**S**TTEDLAKK**F**LDDFNS**E**A**E**NLSH**Q**SS**L**ASWEYNTNISDENVQ
Mustela putorius	MLGSSWLLLSLAALTAAQ**S**TTEDLAK**TF**LE**K**FNY**E**A**E**ELS**YQ**NS**L**ASWNYNTNITDENIQ
M. auratus	MSSSSWLLLSLVAVTTAQ**S**IIEE**Q**AK**TF**LD**K**FNQ**E**A**ED**LS**YQ**SA**L**ASWNYNTNITEENAQ
Felis catus	MSGSFWLLLSFAALTAAQ**S**TTEELAK**TF**LE**K**FN**HE**A**E**ELS**YQ**SS**L**ASWNYNTNITDENVQ
Manis javanica	MSGSSWLLLSLVAVTAAQ**S**TSDEEAK**TF**LE**K**FNS**E**A**E**ELS**YQ**SS**L**ASWNYNTNITDENVQ
Canis lupus	MSGSSWLLLSLAALTAAQ**S**T-EDLVK**TF**LE**K**FNY**E**A**E**ELS**YQ**SS**L**ASWNYNINITDENVQ
Equus caballus	MSGSSWLLLSLVAVTAAQ**S**TTEDLAK**TF**LE**K**FNS**E**A**E**ELSH**Q**SS**L**ASWSYNTNITDENVQ

Sus scrofa	MSGSFWLLLSLIPVTAAQ**S**TTEELAK**TF**LE**K**FNL**E**A**ED**LA**YQ**SS**L**ASWTINTNITDENIQ
Bos taurus	MTGSFWLLLSLVAVTAAQ**S**TTEE**Q**AK**TF**LE**K**FN**HE**A**ED**LS**YQ**SS**L**ASWNYNTNITDENVQ
Ovis aries	MTGSFWLLLSLVAVTAAQ**S**TTEG**Q**AK**TF**LE**K**FN**HE**A**ED**LS**YQ**SS**L**ASWNYNTNITDENVQ
O. cuniculus	MSGSSWLLLSLVAVTAAQ**S**TIEELAK**TF**LE**K**FNQ**E**A**ED**LS**YQ**SA**L**ASWDYNTNITEENVQ
Mus musculus	MSSSSWLLLSLVAVTTAQ**S**LTEENAK**TF**LNNFNQ**E**A**ED**LS**YQ**SS**L**ASWNYNTNITEENAQ
Cricetulus griseus	MSSSSWLLLSLVAVTTAQ**S**IIEE**Q**AK**TF**LD**K**FNQ**E**A**ED**LS**YQ**SA**L**ASWNYNTNITEENAQ
Gallus gallus	MLLHFWLLCGLSAVVTPQDVTQE-AQ**TF**LAEFNVRA**ED**IS**Y**ENS**L**ASWNYNTNITEETAR
	* *** .: .:.:.*. : .:.** .** **:: ::.:**** * **::*. .

Homo sapiens	NMNNAGDKWSAFLKEQST**L**AQ**MY**PLQEIQNLTVKLQLQALQQNGSSVLSEDKSKRLNTIL
Macaca mulatta	NMNNAGEKWSAFLKEQST**L**AQ**MY**PLQEIQNLTVKLQLQALQQNGSSVLSEDKSKRLNTIL
Pan troglodytes	NMNNAGDKWSAFLKEQST**L**AQ**MY**PLQEIQNLTVKLQLQALQQNGSSVLSEDKSKRLNTIL
Rhinolophus sp	KMDEAGAKWSDFYKKQSK**L**AKNFSLEEIHNDTVKLQLQILQQSGSPVLSEDKSKRLNSIL
Mustela putorius	KMNIAGAKWSAFYEEESQHAKT**Y**PLEEIQDPIIKRQLRALQQSGSSVLSADKRERLNTIL
M. auratus	KMNEAAAKWSAFYEEQSK**L**AKN**Y**SLQEVQNLTIKRQLQALQQSGSSALSADKNKQLNTIL
Felis catus	KMNEAGAKWSAFYEEQSK**L**AKT**Y**PLAEIHNTTVKRQLQALQQSGSSVLSADKSQRLNTIL
Manis javanica	KMNVAGAKWSTFYEEQSKIAKN**Y**QLQNIQNDTIKRQLQALQLSGSSALSADKNQRLNTIL
Canis lupus	KMNNAGAKWSAFYEEQSK**L**AKT**Y**PLEEIQDSTVKRQLRALQHSGSSVLSADKNQRLNTIL
Equus caballus	KMNEAGARWSAFYEEQCK**L**AKT**Y**PLEEIQNLTVKRQLQALQQSGSSVLSADKSKRLNEIL
Sus scrofa	KMNDARAKWSAFYEEQSRIAKT**Y**PLDEIQTLILKRQLQALQQSGTSGLSADKSKRLNTIL
Bos taurus	KMNEARAKWSAFYEEQSRMAKT**Y**SLEEIQNLTLKRQLKALQHSGTSALSAEKSKRLNTIL
Ovis aries	KMNEARAKWSAFYEEQSRMART**Y**SLEEIQNLTLKRQLKALQHSGTSVLSAEKSKRLNTIL
O. cuniculus	KMNDAEAKWSAFYEEQSK**L**AKT**Y**PSQEVQNLTVKRQLQALQQSGSSALSADKSKQLNTIL
Mus musculus	KMSEAAAKWSAFYEEQSKTAQSFSLQEIQTPIIKRQLQALQQSGSSALSADKNKQLNTIL
Cricetulus griseus	KMNEAAAKWSAFYEEQSK**L**AKN**Y**SLQEVQNLIIKRQLQALQQSGSSALSADKNKQLNTIL
Gallus gallus	KMSEAGAKWAAFYEEASRNASRFSLANIQDAVTRLQIQSLQDRGSSVLSPEKYSRLNSVM
	:*. * .*: * :: . * : ::: . *:. ** *:. ** :* ..** ::

Homo sapiens	NTMSTIYSTGKVCNPDNPQECLLLEPGLNEIMANSLDYNERLWAWESWRSEVGKQLRPLY
Macaca mulatta	NTMSTIYSTGKVCNPNNPQECLLLDPGLNEIMEKSLDYNERLWAWEGWRSEVGKQLRPLY

```
Pan troglodytes     NTMSAIYSTGKVCNPNNPQECLLLEPGLNEIMANSLDYNERLWAWESWRSEVGKQLRPLY

Rhinolophus sp      NAMSTIYSTGKVCKPNNPQECLLLEPGLDNIMETSKDYNERLWAWEGWRAEVGKQLRPLY

Mustela putorius    NAMSTIYSTGKACNPNNPQECLLLEPGLDDIMENSKDYNERLWAWEGWRSEVGKQLRPLY

M. auratus          NTMSTIYSTGKVCNPKNPQECLLLEPGLDDIMATSTDYNERLWAWEGWRAEVGKQLRPLY

Felis catus         NAMSTIYSTGKACNPNNPQECLLLEPGLDDIMENSKDYNERLWAWEGWRAEVGKQLRPLY

Manis javanica      NTMSTIYSTGKVCNPGNPQECSLLEPGLDNIMESSKDYNERLWAWEGWRSEVGKQLRPLY

Canis lupus         NSMSTVYSTGKACNPSNPQECLLLEPGLDDIMENSKDYNERLWAWEGWRSEVGKQLRPLY

Equus caballus      NTMSTIYSTGKVCNPSNPQECLLLEPGLDAIMENSKDYNQRLWAWEGWRSEVGKQLRPLY

Sus scrofa          NTMSTIYSSGKVLDPNNPQECLVLEPGLDEIMENSKDYSRRLWAWESWRAEVGKQLRPLY

Bos taurus          NKMSTIYSTGKVLDP-NTQECLALEPGLDDIMENSRDYNRRLWAWEGWRAEVGKQLRPLY

Ovis aries          NKMSTIYSTGKVLDP-NTQECLALEPGLDDIMENSRDYNRRLWAWEGWRAEVGKQLRPLY

O. cuniculus        STMSTIYSTGKVCNQSNPQECFLLEPGLDEIMAKSTDYNERLWAWEGWRSVVGKQLRPLY

Mus musculus        NTMSTIYSTGKVCNPKNPQECLLLEPGLDEIMATSTDYNSRLWAWEGWRAEVGKQLRPLY

Cricetulus griseus  NTMSTIYSTGKVCNPKNPQECLLLEPGLDDIMATSTDYNERLWAWEGWRAEVGKQLRPLY

Gallus gallus       NSMSTIYSTGVVCKATEPFDCLVLEPGLDDIMANSIDYHERLWAWEGWRADVGRMMRPLY

                    . **::**:* . .  :. :*  *:***: ** .* **  ******.**: **. :****


Homo sapiens        EEYVVLKNEMARANHYEDYGDYWRGDYEVNGVDGYDYSRGQLIEDVEHTFEEIKPLYEHL

Macaca mulatta      EEYVVLKNEMAGANHYKDYGDYWRGDYEVNGVDGYDNNRDQLIEDVERTFEEIKPLYEHL

Pan troglodytes     EEYVVLKNEMARANHYEDYGDYWRGDYEVNGVDGYDYSRGQLIEDVEHTFEEIKPLYEHL

Rhinolophus sp      EEYVVLKNEMARGYHYEDYGDYWRRDYETEGSPDLEYSRDQLIKDVERIFAEIKPLYEQL

Mustela putorius    EEYVALKNEMARANNYEDYGDYWRGDYEEEWADGYSYSRNQLIEDVEHTFTQIKPLYEHL

M. auratus          EEYVVLKNEMARANNYEDYGDYWRGDYEAEGADGYNYNGNQLIEDVERTFKEIKPLYEQL

Felis catus         EEYVALKNEMARANNYEDYGDYWRGDYEEEWTDGYNYSRSQLIKDVEHTFTQIKPLYQHL

Manis javanica      EEYVVLKNEMARANHYEDYGDYWRGDYEAEGANGYNYSRDHLIEDVEHIFTQIKPLYEHL

Canis lupus         EEYVALKNEMARANNYEDYGDYWRGDYEEEWENGYNYSRNQLIDDVELTFTQIMPLYQHL

Equus caballus      EEYVVLKNEMARANNYEDYGDYWRGDYEAEGPSGYDYSRDQLIEDVERTFAEIKPLYEHL

Sus scrofa          EEYVVLENEMARANNYEDYGDYWRGDYEVTGTGDYDYSRNQLMEDVERTFAEIKPLYEHL

Bos taurus          EEYVVLENEMARANNYEDYGDYWRGDYEVTGAGDYDYSRDQLMKDVERTFAEIKPLYEQL

Ovis aries          EEYVVLENEMARANNYEDYGDYWRGDYEVTGAGDYDYSRDQLMKDVERTFEEIKPLYEQL
```

O. cuniculus   EEYVVLKNEMARANNYEDYGDYWRADYEAEGADGYDYSRSQLIDDVERTFSEIKPLYEQL

Mus musculus   EEYVVLKNEMARANNYNDYGDYWRGDYEAEGADGYNYNRNQLIEDVERTFAEIKPLYEHL

Cricetulus griseus EEYVVLKNEMARANNYKDYGDYWRGDYEAEGADGYNYNGNQLIEDVERTFKEIKPLYEQL

Gallus gallus   EEYVELKNEAARLNNYSDYGDYWRANYETDYPEEYKYSRDQLVQDVEKTFEQIKPLYQHL

**** *:** *   :*.******* :**   . . .:*:.*** * :* ***::*

Homo sapiens   HAYVRAKLMNAYPS-YISPIGCLPAHLLGDMWGRFWTNLYSLTVPFGQKPNIDVTDAMVD

Macaca mulatta   HAYVRAKLMNAYPS-YISPTGCLPAHLLGDMWGRFWTNLYSLTVPFGQKPNIDVTDAMVN

Pan troglodytes   HAYVRAKLMNAYPS-YISPIGCLPAHLLGDMWGRFWTNLYSLTVPFGQKPNIDVTDAMVD

Rhinolophus sp   HAYVRTKLMDTYPF-HISPTGCLPAHLLGDMWGRFWTNLYPLTVPFGQKPNIDVTDAMLN

Mustela putorius   HAYVRAKLMDAYPS-RISPTGCLPAHLLGDMWGRFWTNLYPLMVPFRQKPNIDVTDAMVN

M. auratus   HAYVRTKLMNTYPS-YISPTGCLPAHLLGDMWGRFWTNLYPLTVPFGQKPNIDVTDAMVN

Felis catus   HAYVRAKLMDTYPS-RISPTGCLPAHLLGDMWGRFWTNLYPLTVPFGQKPNIDVTDAMVN

Manis javanica   HAYVRAKLMDNYPS-HISPTGCLPAHLLGDMWGRFWTNLYPLTVPFRQKPNIDVTDAMVN

Canis lupus   HAYVRTKLMDTYPS-YISPTGCLPAHLLGDMWGRFWTNLYPLTVPFGQKPNIDVTNAMVN

Equus caballus   HAYVRAKLMDTYPS-HINPTGCLPAHLLGDMWGRFWTNLYSLTVPFGQKPNIDVTDAMVD

Sus scrofa   HAYVRAKLMDAYPS-RISPTGCLPAHLLGDMWGRFWTNLYPLTVPFGEKPSIDVTEAMVN

Bos taurus   HAYVRAKLMHTYPS-YISPTGCLPAHLLGDMWGRFWTNLYSLTVPFEHKPSIDVTEKMEN

Ovis aries   HAYVRAKLMDTYPS-YISPTGCLPAHLLGDMWGRFWTNLYSLTVPFEHKPSIDVTEKMKN

O. cuniculus   HAFVRTKLMDAYPS-RISPTGCLPAHLLGDMWGRFWTNLYSLTVPFGQKPNIDVTDTMVN

Mus musculus   HAYVRRKLMDTYPS-YISPTGCLPAHLLGDMWGRFWTNLYPLTVPFAQKPNIDVTDAMMN

Cricetulus griseus HAYVRTKLMDTYPS-YISPTGCLPAHLLGDMWGRFWTNLYPLTVPFGQKPNIDVTDAMVN

Gallus gallus   HAYVRHRLEQVYGSELINPTGCLPAHLLGDMWGRFWTNLYNLTVPYPEKPNIDVTSAMAQ

: .*   *   *.* ******************** * **: **.****. * :

Homo sapiens   QAWDAQRIFKEAEKFFVSVGLPNMT**Q**GFW**EN**SMLTDPGNVQKAVCHPTAWDLG**KGD**F**R**IL

Macaca mulatta   QAWNAQRIFKEAEKFFVSVGLPNMT**Q**GFW**EN**SMLTDPGNVQKVVCHPTAWDLG**KGD**F**R**II

Pan troglodytes   QAWDAQRIFKEAEKFFVSVGLPNMT**Q**GFW**EN**SMLTDPGNVQKAVCHPTAWDLG**KGD**F**R**IL

Rhinolophus sp   QNWDAKRIFKEAEKFFVSIGLPNMTEGFWN**N**SMLTDPGDGRKVVCHPTAWDLG**KGD**F**R**IK

Mustela putorius   QSWDARRIFEEAETFFVSVGLPNMTEGFWQ**N**SMLTEPGDNRKVVCHPTAWDLG**K**R**D**F**R**IK

```
M. auratus          QGWNAERIFKEAEKFFVSVGLPYMTQGFWENSMLTDPGDDRKVVCHPTAWDLGKGDFRIK

Felis catus         QSWDARRIFKEAEKFFVSVGLPNMTQGFWENSMLTEPGDSRKVVCHPTAWDLGKGDFRIK

Manis javanica      QTWDANRIFKEAEKFFVSVGLPKMTQTFWENSMLTEPGDGRKVVCHPTAWDLGKHDFRIK

Canis lupus         QSWDARKIFKEAEKFFVSVGLPNMTQEFWGNSMLTEPSDSRKVVCHPTAWDLGKGDFRIK

Equus caballus      QSWDAKRIFEEAEKFFVSVGLPNMTQGFWENSMLTEPGDGRKVVCHPTAWDLGKGDFRIK

Sus scrofa          QSWDAIRIFEEAEKFFVSIGLPNMTQGFWNNSMLTEPGDGRKVVCHPTAWDLGKGDFRIK

Bos taurus          QSWDAERIFKEAEKFFVSISLPYMTQGFWDNSMLTEPGDGRKVVCHPTAWDLGKGDFRIK

Ovis aries          QSWDAERIFKEAEKFFVSIGLPYMTQGFWDNSMLTEPGDGRKVVCHPTAWDLGKGDFRIK

O. cuniculus        QGWDAERIFKEAEKFFVSVGLPSMTQGFWENSMLTEPGDGRKVVCHPTAWDLGKGDFRIK

Mus musculus        QGWDAERIFQEAEKFFVSVGLPHMTQGFWANSMLTEPADGRKVVCHPTAWDLGHGDFRIK

Cricetulus griseus  QGWDAERIFKEAEKFFVSVGLPHMTQGFWGNSMLTDPGDDRKVVCHPTAWDLGKGDFRIK

Gallus gallus       KNWDAMKIFKTAEAFFASIGLYNMTEGFWTNSMLTEPTDNRKVVCHPTAWDMGKNDYRIK

                    : *:* .**: ** **.*:.*  **: ** *****:* : .*.********:*: *:**


Homo sapiens        MCTKVTMDDFLTAHHEMGHIQYDMAYAAQPFLLRNGANEGFHEAVGEIMSLSAATPKHLK

Macaca mulatta      MCTKVTMDDFLTAHHEMGHIQYDMAYAAQPFLLRNGANEGFHEAVGEIMSLSAATPKHLK

Pan troglodytes     MCTKVTMDDFLTAHHEMGHIQYDMAYAAQPFLLRNGANEGFHEAVGEIMSLSAATPKHLK

Rhinolophus sp      MCTKVTMEDFLTAHHEMGHIQYDMAYASQPYLLRNGANEGFHEAVGEVMSLSVATPKHLK

Mustela putorius    MCTKVTMDDFLTAHHEMGHIQYDMAYAEQPFLLRNGANEGFHEAVGEIMSLSAATPNHLK

M. auratus          MCTKVTMDNFLTAHHEMGHIQYDMAYATQPFLLRNGANEGFHEAVGEIMSLSAATPEHLK

Felis catus         MCTKVTMDDFLTAHHEMGHIQYDMAYAVQPFLLRNGANEGFHEAVGEIMSLSAATPNHLK

Manis javanica      MCTKVTMDDFLTAHHEMGHIQYDMAYAMQPYLLRNGANEGFHEAVGEIMSLSAATPKHLK

Canis lupus         MCTKVTMDDFLTAHHEMGHIQYDMAYAAQPFLLRNGANEGFHEAVGEIMSLSAATPNHLK

Equus caballus      MCTKVTMDDFLTAHHEMGHIQYDMAYAVQPYLLRNGANEGFHEAVGEIMSLSAATPNHLK

Sus scrofa          MCTKVTMDDFLTAHHEMGHIQYDMAYAIQPYLLRNGANEGFHEAVGEIMSLSAATPHYLK

Bos taurus          MCTKVTMDDFLTAHHEMGHIQYDMAYAAQPYLLRNGANEGFHEAVGEIMSLSAATPHYLK

Ovis aries          MCTKVTMDDFLTAHHEMGHIQYDMAYATQPYLLRNGANEGFHEAVGEIMSLSAATPHYLK

O. cuniculus        MCTKVTMDNFLTAHHEMGHIQYDMAYATQPFLLRNGANEGFHEAVGEIMSLSAATPEHLK

Mus musculus        MCTKVTMDNFLTAHHEMGHIQYDMAYARQPFLLRNGANEGFHEAVGEIMSLSAATPKHLK

Cricetulus griseus  MCTKVTMDNFLTAHHEMGHIQYDMAYATQPFLLRNGANEGFHEAVGEIMSLSAATPKHLK
```

```
Gallus gallus        MCTKVTMDDFLTAHHEMGHIEYDMAYSVQPFLLRNGANEGFHEAVGEIMSLSAATPQHLK
                     *******::***********:*****: **:****************:****.*** :**


Homo sapiens         SIGLLSPDFQEDNETEINFLLKQALTIVGTLPFTYMLEKWRWMVFKGEIPKDQWMKKWWE
Macaca mulatta       SIGLLSPDFQEDNETEINFLLKQALTIVGTLPFTYMLEKWRWMVFKGEIPKDQWMKKWWE
Pan troglodytes      SIGLLSPDFQEDNETEINFLLKQALTIVGTLPFTYMLEKWRWMVFKGEIPEDQWMKKWWE
Rhinolophus sp       TMGLLSSDFLEDNETEINFLFKQALNIVGTLPFTYMLEKWRWMVFKGEIPKEEWMKKWWE
Mustela putorius     NIGLLPPDFSEDSETDINFLLKQALTIVGTLPFTYMLEKWRWMVFKGEIPKEQWMQKWWE
M. auratus           SIGLLPSDFQEDNETEINFLLKQALTIVGTLPFTYMLEKWRWMVFKGDIPKEQWMEKWWE
Felis catus          TIGLLSPGFSEDSETEINFLLKQALTIVGTLPFTYMLEKWRWMVFKGEIPKEQWMQKWWE
Manis javanica       NIGLLPPDFYEDNETEINFLLKQALTIVGTLPFTYMLEKWRWMVFSGQIPKEQWMKKWWE
Canis lupus          NIGLLPPSFFEDSETEINFLLKQALTIVGTLPFTYMLEKWRWMVFKGEIPKDQWMKTWWE
Equus caballus       AIGLLPPDFYEDSETEINFLLKQALTIVGTLPFTYMLEKWRWMVFKGEIPKEEWMKKWWE
Sus scrofa           ALGLLPPDFYEDSETEINFLLKQALTIVGTLPFTYMLEKWRWMVFKGEIPKEQWMQKWWE
Bos taurus           ALGLLAPDFHEDNETEINFLLKQALTIVGTLPFTYMLEKWRWMVFKGEIPKQQWMEKWWE
Ovis aries           ALGLLAPDFYEDNETEINFLLKQALTIVGTLPFTYMLEKWRWMVFKGEIPKQQWMEKWWE
O. cuniculus         SIGLLPYDFHEDNETEINFLLKQALTIVGTLPFTYMLEKWRWMVFKGEIPKEQWMQKWWE
Mus musculus         SIGLLPSDFQEDSETEINFLLKQALTIVGTLPFTYMLEKWRWMVFRGEIPKEQWMKKWWE
Cricetulus griseus   SIGLLPSNFHEDNETEINFLLKQALTIVGTLPFTYMLEKWRWMVFKGDIPKEKWMEKWWE
Gallus gallus        SLDLLEPTFQEDEETEINFLLKQALTIVGTMPFTYMLEKWRWMVFNGEITKQEWTKRWWK
                      :.**   * **.**:****:****.****:************** *:*.:::* : **:


Homo sapiens         MKREIVGVVEPVPHDETYCDPASLFHVSNDYSFIRYYTRTLYQFQFQEALCQAAKHEGPL
Macaca mulatta       MKREIVGVVEPVPHDETYCDPASLFHVSNDYSFIRYYTRTLYQFQFQEALCQAAKHEGPL
Pan troglodytes      MKREIVGVVEPVPHDETYCDPASLFHVSNDYSFIRYYTRTLYQFQFQEALCQAAKHEGPL
Rhinolophus sp       MKRKIVGVVEPVPHDETYCDPASLFHVANDYSFIRYYTRTIFEFQFHEALCRIAKHDGPL
Mustela putorius     MKRDIVGVVEPLPHDETYCDPAALFHVANDYSFIRYYTRTIYQFQFQEALCQIAKHEGPL
M. auratus           MKREIVGVVEPLPHDETYCDPAALFHVSNDYSFIRYYTRTIYQFQFQEALCQAAKHDGPL
Felis catus          MKREIVGVVEPVPHDETYCDPASLFHVANDYSFIRYYTRTIYQFQFQEALCRIAKHEGPL
Manis javanica       MKREIVGVVEPVPHDETYCDPASLFHVANDYSFIRYYTRTIYQFQFQEALCQTAKHEGPL
```

```
Canis lupus         MKRNIVGVVEPVPHDETYCDPASLFHVANDYSFIRYYTRTIYQFQFQEALCQIAKHEGPL

Equus caballus      MKREIVGVVEPVPHDETYCDPAALFHVANDYSFIRYYTRTIYQFQFQEALCQTAKHEGPL

Sus scrofa          MKREIVGVVEPLPHDETYCDPACLFHVAEDYSFIRYYTRTIYQFQFHEALCRTAKHEGPL

Bos taurus          MKREIVGVVEPLPHDETYCDPACLFHVAEDYSFIRYYTRTIYQFQFHEALCKTAKHEGAL

Ovis aries          MKREIVGVVEPLPHDETYCDPACLFHVAEDYSFIRYYTRTIYQFQFHEALCKTAKHEGAL

O. cuniculus        MKREIVGVVEPMPHDETYCDPAALFHVANDYSFIRYYTRTIYQFQFQEALCQAAQHEGPL

Mus musculus        MKREIVGVVEPLPHDETYCDPASLFHVSNDYSFIRYYTRTIYQFQFQEALCQAAKYNGSL

Cricetulus griseus  MKREIVGVVEPLPHDETYCDPAALFHVSNDYSFIRYYTRTIYQFQFQEALCQAAKHDGPL

Gallus gallus       MKREIVGVVEPVPHDETYCDPAALFHVANDYSFIRYYTRTIYQFQFQEALCKAANHTGPL

                    ***.*******:**********.****::***********:::***:****. *:: *.*


Homo sapiens        HKCDISNSTEAGQKLFNMLRLGKSEPWTLALENVVGAKNMNVRPLLNYFEPLFTWLKDQN Macaca
mulatta      HKCDISNSTEAGQKLLNMLKLGESEPWTLALENVVGAKNMNVRPLLNYFEPLFTWLKDQN

Pan troglodytes     HKCDISNSTEAGQKLFNMLRLGKSEPWTLALENVVGAKNMNVRPLLNYFEPLFTWLKDQN

Rhinolophus sp      HKCDISNSTDAGEKLHQMLSVGKSQPWTSVLKDFVGSKNMDVGPLLRYFEPLYTWLTEQN

Mustela putorius    YKCDISNSSEAGQKLHEMLSLGRSKPWTFALERVVGAKTMDVRPLLNYFEPLFTWLKEQN

M. auratus          HKCDISNSTEAGQKLLNMLRLGKSEPWTLALENVVGARNMDVRPLLNYFEPLSVWLKEQN

Felis catus         HKCDISNSSEAGKKLLQMLTLGKSKPWTLALEHVVGEKKMNVTPLLKYFEPLFTWLKEQN

Manis javanica      HKCDISNSAEAGQKLLQMLSLGKSKPWTLALERVVGTKNMDVRPLLNYFEPLLTWLKEQN

Canis lupus         HKCDISNSSEAGQKLLEMLKLGKSKPWTYALEIVVGAKNMDVRPLLNYFEPLFTWLKEQN

Equus caballus      HKCDISNSTEAGQKLLQMLSLGKSEPWTLALERIVGVKNMDVRPLLNYFEPLFTWLKDQN

Sus scrofa          YKCDISNSTEAGQKLLQMLSLGKSEPWTLALENIVGVKTMDVKPLLSYFEPLLTWLKAQN

Bos taurus          FKCDISNSTEAGQRLLQMLRLGKSEPWTLALENIVGIKTMDVKPLLNYFEPLFTWLKEQN

Ovis aries          FKCDISNSTEAGQRLLQMLRLGKSEPWTLALENIVGIKTMDVKPLLNYFEPLFTWLKEQN

O. cuniculus        HKCDISNSTEAGQKLLNMLRLGRSEPWTLALENVVGAKNMDVRPLLNYFEPLFTWLKEQN

Mus musculus        HKCDISNSTEAGQKLLKMLSLGNSEPWTKALENVVGARNMDVKPLLNYFQPLFDWLKEQN

Cricetulus griseus      HKCDISNSTEAGQKLLNMLRLGKSEPWTLALENVVGARNMDVRPLLNYFEPLSVWLKEQN

Gallus gallus       HKCDITNSTAAGGNLRQLLELGKSKPWTQALESATGEKYMNATPLLHYFEPLFNWLQKNN

                    .****:**: ** .* ::* :* *:*** .*:  .* . *:. *** **:**  **  :*


Homo sapiens        KNSFVGWSTDWSPYADQSIKVRISLKSALGDKAYEWNDNEMYLFRSSVAYAMRQYFLKVK
```

```
Macaca mulatta      KNSFVGWSTDWSPYADQSIKVRISLKSALGDKAYEWNDNEMYLFRSSVAYAMRTYFLEIK

Pan troglodytes     KNSFVGWSTDWSPYADQSIKVRISLKSALGDKAYEWNDNEMYLFRSSVAYAMRQYFLKVK

Rhinolophus sp      RKSFVGWNTDWSPYADQSIKVRISLKSALGEKAYEWNNNEMYLFRSSVAYAMREYFLKTK

Mustela putorius    RNSFVGWNTDWSPYADQSIKVRISLKSALGEKAYEWNDNEMYFFQSSIAYAMREYFSKVK

M. auratus          KNSFVGWNTDWSPYADQSIKVRISLKSALGENAYEWDDNEMYLFRASVAYAMRVYFAKNK

Felis catus         RNSFVGWNTDWRPYADQSIKVRISLKSALGDEAYEWNDNEMYLFRSSVAYAMREYFSKVK

Manis javanica      KNSFVGWNTDWSPYAAQSIKVRISLKSALGEKAYEWNDSEMYLFRSSVAYAMREYFSKVK

Canis lupus         RNSFVGWNTDWSPYADQSIKVRISLKSALGEKAYEWNNNEMYLFRSSIAYAMRQYFSEVK

Equus caballus      KNSFVGWSTNWSPYADQSIKVRISLKSALGEKSYEWNDNEMYLFQSSVAYAMRVYFLKAK

Sus scrofa          GNSSVGWNTDWTPYADQSIKVRISLKSALGEDAYEWNDNEMYLFRSSIAYAMRNYFSSAK

Bos taurus          RNSFVGWSTEWTPYSDQSIKVRISLKSALGENAYEWNDNEMYLFQSSVAYAMRKYFSEAR

Ovis aries          RNSFVGWSTEWTPYSDQSIKVRISLKSALGENAYEWNDNEMYLFRSSVAYAMRKYFLKER

O. cuniculus        RNSFVGWSTEWTPYADQSIKVRISLKTALGDQAYEWNDSEMYLFRSSVAYAMRKYFSEVK

Mus musculus        RNSFVGWNTEWSPYADQSIKVRISLKSALGANAYEWTNNEMFLFRSSVAYAMRKYFSIIK

Cricetulus griseus  KNSFVGWNTDWSPYADQSIKVRISLKSALGENAYEWNDNEMYLFRATVAYAMRVYFAKNK

Gallus gallus       SGRSIGWNTDWTPYSDNAIKVRISLKAALGDDAYVWDASELFLFKSSIAYAMRKYFAKEK

                        :**.*:* **: ::********:*** .:* *  .*:::*.:::***** **   .


Homo sapiens        NQMILFGEEDVRVANLKPRISFNFFVTAPKNVSDIIPRTEVEKAIRMSRSRINDAFRLND

Macaca mulatta      HQTILFGEEDVRVADLKPRISFNFYVTAPKNVSDIIPRTEVEEAIRISRSRINDAFRLND

Pan troglodytes     NQMILFGEEDVRVANLKPRISFNFFVTAPKNVSDIIPRTEVEKAIRKSRSRINDAFRLND

Rhinolophus sp      NQTILFGEEDVWVSNLKPRISFNFYVTSPRNLSDIIPRPEVEGAIRMSRSRINDAFRLDD

Mustela putorius    NQTIPFVGKDVRVSDLKPRISFNFIVTSPENMSDIIPRADVEEAIRKSRGRINDAFRLDD

M. auratus          TQTVPFGVEDIRVSDLKPRVSFNFFVTSPQNVSDIIPRNEVEEAVRLSRGRINDVFGLDD

Felis catus         NQTIPFVEDNVWVSNLKPRISFNFFVTASKNVSDVIPRSEVEEAIRMSRSRINDAFRLDD

Manis javanica      KQTIPFEDECVRVSDLKPRVSFIFFVTLPKNVSAVIPRAEVEEAIRISRSRINDAFRLDD

Canis lupus         NQTIPFVEDNVWVSDLKPRISFNFSVTSPGNVSDIIPRTEVEEAIRMYRSRINDVFRLDD

Equus caballus      NQTILFGEEDVWVSDLKPRISFNFFVTSPKNASDIIPRTDVEEAIRMSRSRINDAFRLDD

Sus scrofa          NETIPFGAVDVWVSDLKPRISFNFFVTSPANMSDIIPRSDVEKAISMSRSRINDAFRLDD

Bos taurus          NETVLFGEDNVWVSDKKPRISFKFFVTSPNNVSDIIPRTEVENAIRLSRDRFNDVFQLDD
```

```
Ovis aries          NETIPFGEENVWVSDKKPRISFKFFVTSPNNVSDIIPRTEVENAIRLCRDRINDAFQLDD

O. cuniculus        NQTILFGEEDVRVSDLKPRISFNFFVTAPNNVNDIIPRNEVEEAISMSRSRINDIFRLDD

Mus musculus        NQTVPFLEEDVRVSDLKPRVSFYFFVTSPQNVSDVIPRSEVEDAIRMSRGRINDVFGLND

Cricetulus griseus  TQTVLFGVEDIRVSDLKPRVSFNFFVTSPQNVSDIIPRNEVEEAVRFSRGRINDVFGLDD

Gallus gallus       EQNVDFQVTDIHVGEETQRVSFYLTVSMPGNVSDIVPRADVEKAIRMSRGRISEAFRLDD

                     : : *    :.*.: . *:** : *: . * . ::** :** *:   *.*:.: * *:*


Homo sapiens        NSLEFLGIQPTLGPPNQPPVSIWLIVFGVVMGVIVVGIVILIFTGIRDRKKKNKARSGE-

Macaca mulatta      NSLEFLGIQTTLAPPYQSPVTTWLIVFGVVMGVIVAGIVVLIFTGIRDRKKKNQARSEE-

Pan troglodytes     NSLEFLGIQPTLGPPNQPPVSIWLIVFGVVMGVIVVGIVILIFTGIRDRKKKNKARSEE-

Rhinolophus sp      NSLEFLGIQPTLGPPYQPPVTIWLIVFGVVMAVVVVGIVVLIITGIRDRRKKDQARSEE-

Mustela putorius    NSLEFLGIQPTLEPPYQPPVTIWLIVFGVVMGVVVVGIFLLIFSGIRNRRKNNQARSEE-

M. auratus          NSLEFLGINPTLSPPYQPPVTIWLIIFGVVMGIVVVGIIILIFTGIKGRKKKNETKREE-

Felis catus         NSLEFLGIQPTLSPPYQPPVTIWLIVFGVVMGVVVVGIVLLIVSGIRNRRKNNQARSEE-

Manis javanica      NSLEFLGIQPTLQPPYQPPVTIWLIVFGVVMGVVVVGIVVLIFTGIRDRKKKDQARSEQ-

Canis lupus         NSLEFLGIQPTPGPPYEPPVTIWLIVFGVVMGVVVVGIVLLIFSGIRNRRKNDQARGEE-

Equus caballus      NTLEFLGIQPTLGPPYQPPVTVWLIAFGVVMGLVVVGIVVLIATGIRGRRKKNQARSEE-

Sus scrofa          NTLEFLGIQPTLGPPDEPPVTVWLIIFGVVMGLVVVGIVVLIFTGIRDRRKKKQASSEE-

Bos taurus          NSLEFLGIQPTLGPPYEPPVTIWLIIFGVVMGVVVIGIVVLIFTGIRNRRKKNQASSEE-

Ovis aries          NSLEFLGIQPTLRPPYEPPVTIWLIIFGVVMGVVVIGIVVLIFTGIRDQRKKNQASSEE-

O. cuniculus        NSLEFVGIQPTLEPPYESPVPIWLVVFGVVMGMIVIGIVVLIFTGIKDRRKQKQAKREE-

Mus musculus        NSLEFLGIHPTLEPPYQPPVTIWLIIFGVVMALVVVGIIILIVTGIKGRKKKNETKREE-

Cricetulus griseus  NSLEFLGINPTLAPPYQPPVTIWLIIFGVVMGIVVVGIVILIVTGIRARKKNNEAKREE-

Gallus gallus       NTLEFDGIVPTLATPYKPPVTIWLILFGVVMSLIVIGVIVLIITGQRDKRKKARGRANEA

                    *:*** ** .*  .* :.**. **: *****.::* *:.:** :* . ..*:      :


Homo sapiens        ------NPYASIDISKGENNPGFQNTDDVQTSF

Macaca mulatta      ------NPYASIDINKGENNPGFQNTDDVQTSF

Pan troglodytes     ------NPYASVDTSKGENNPGFQNTDDVQTSF

Rhinolophus sp      ------NPYSSVDLSKGENNPGFQNGDDVQTSF
```

```
Mustela putorius    ------NPYASVDLSKGENNPGFQNVDDVQTSF

M. auratus          ------NPYDSVDIGKGESNAGFLSNDDAQTSF

Felis catus         ------NPYASVDLSKGENNPGFQHADDVQTSF

Manis javanica      ------NPYASVDLSKGENNPGFQNVDDVQTSF

Canis lupus         ------NPYASVDLSKGENNPGFQSGDDVQTSF

Equus caballus      ------NPYASVDLSKGENNPGFQNGDDVQTSF

Sus scrofa          ------NPYGSMDLSKGESNSGFQNGDDIQTSF

Bos taurus          ------NPYGSVDLNKGENNSGFQNIDDVQTSL

Ovis aries          ------NPYGSVDLNKGENNSGFQNTDDVQTSL

O. cuniculus        ------NPYGFVDMSKGENNSGFQNSDDIQTSF

Mus musculus        ------NPYDSMDIGKGESNAGFQNSDDAQTSF

Cricetulus griseus  ------NPYDSVDIGKGESNAGFQSNDDVQTSF

Gallus gallus       GSNCEVNPYD----EDGRSNKGFEQSEETQTSF

                          ***      .* .* **   :: ***:
```

1.1 Supplementary Figure 1

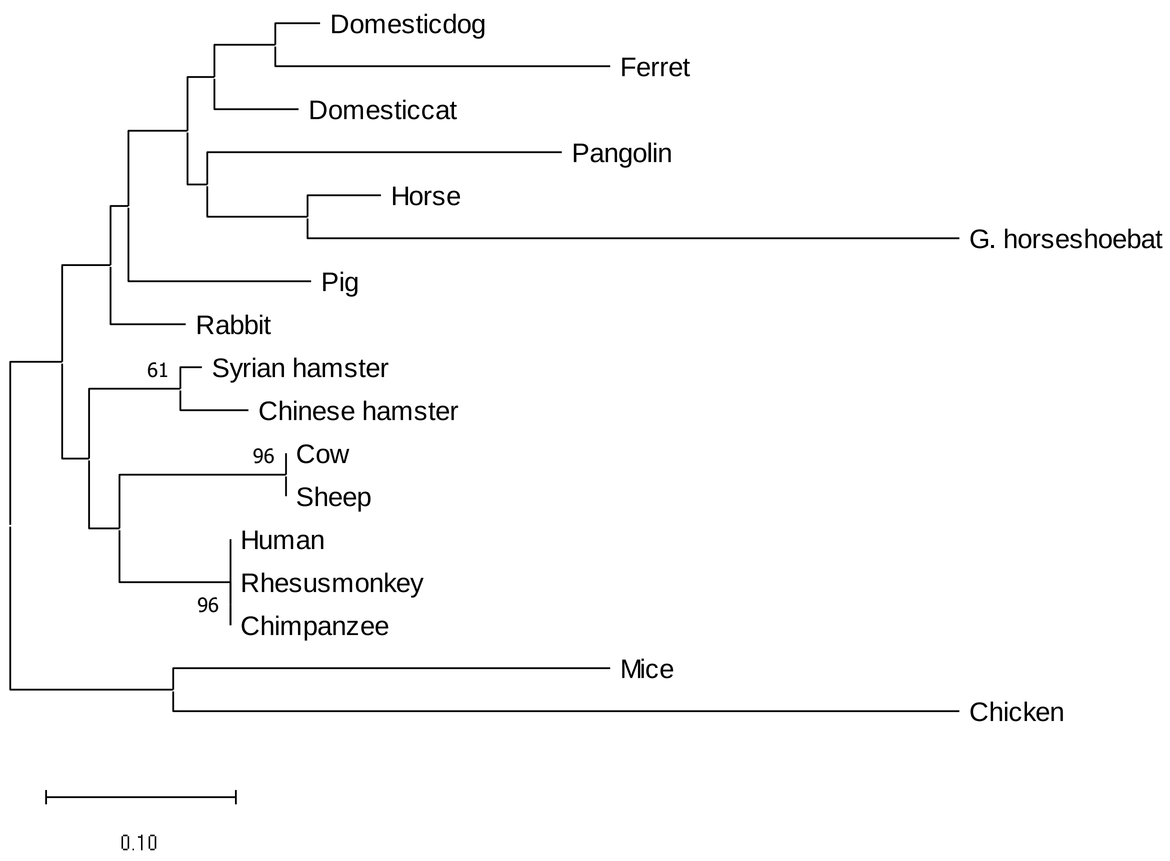

Supplementary Figure 1. Phylogenetic tree of the angiotensin-converting enzyme - 2 (ACE-2) protein sequence for aa positions shown in Table 1 of different animals and humans inferred using the Neighbor-Joining method. The optimal tree with the sum of branch length = 2.16064957 is shown. The percentage of replicate trees in which the associated taxa clustered together in the bootstrap test (1000 replicates) are shown next to the branches. The tree is drawn to scale, with branch lengths in the same units as those of the evolutionary distances used to infer the phylogenetic tree. The evolutionary distances were computed using the Poisson correction method and are in the units of the number of amino acid substitutions per site. This analysis involved 17 amino acid sequences. All ambiguous positions were removed for each sequence pair (pairwise deletion option). There were a total of 22 positions in the final dataset. Evolutionary analyses were conducted in MEGA X from protein sequence alignment depicted in Supplementary Data.
